# Capillary Torque on a Particle Rotating at an Interface

**DOI:** 10.1021/acs.langmuir.1c00851

**Published:** 2021-06-11

**Authors:** Abhinav Naga, Doris Vollmer, Hans-Jürgen Butt

**Affiliations:** Max Planck Institute for Polymer Research, Ackermannweg 10, 55128 Mainz, Germany

## Abstract

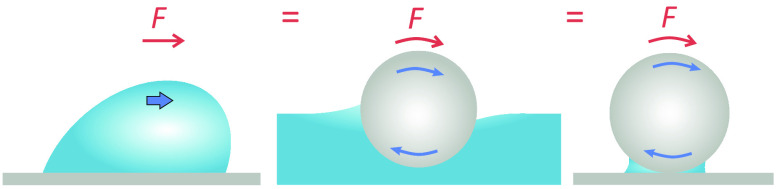

Small particles attach to liquid–fluid interfaces due to capillary forces. The influence of rotation on the capillary force is largely unexplored, despite being relevant whenever particles roll at a liquid–fluid interface or on a moist solid. Here, we demonstrate that due to contact angle hysteresis, a particle needs to overcome a resistive capillary torque to rotate at an interface. We derive a general model for the capillary torque on a spherical particle. The capillary torque is given by *M* = γ*RLk*(cos Θ_R_ – cos Θ_A_), where γ is the interfacial tension, *R* is the radius of the particle, *L* is the diameter of the contact line, *k* = 24/π^3^ is a geometrical constant, and Θ_R_ and Θ_A_ are the receding and advancing contact angles, respectively. The expression for the capillary torque (normalized by the radius of the particle) is equivalent to the expression for the friction force that a drop experiences when moving on a flat surface. Our theory predicts that capillary torque reduces the mobility of wet granular matter and prevents small (nano/micro) particles from rotating when they are in Brownian motion at an interface.

## Introduction

**Figure 1:**
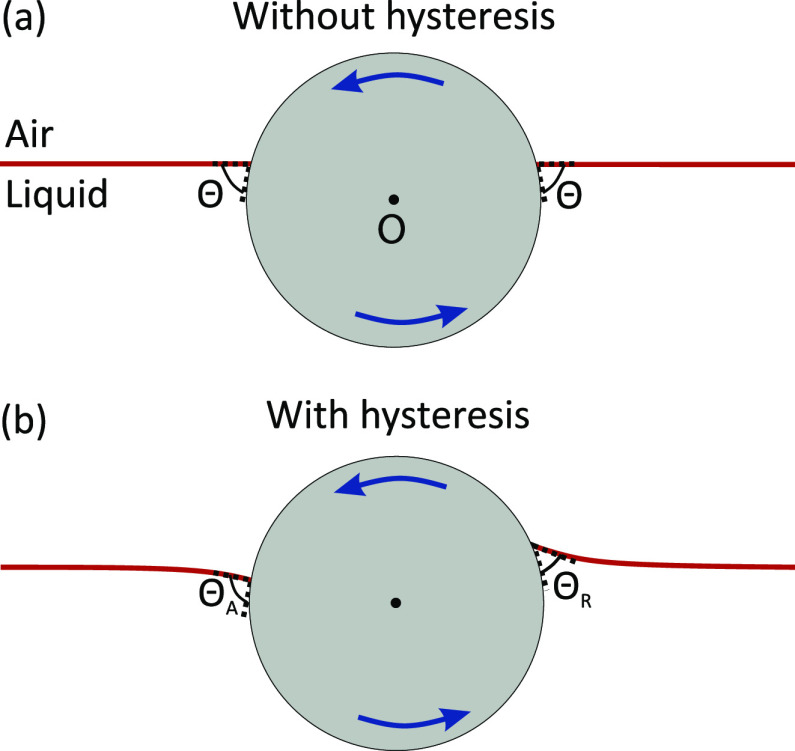
Particle (gray) rotating at a liquid–air interface (red), ignoring gravitational effects. (a) Without contact angle hysteresis, the interface remains flat and symmetric since the contact angle has a unique value, Θ. (b) With contact angle hysteresis, the interface becomes asymmetric. On the right, the contact angle is equal to the receding angle, Θ_R_, whereas on the left, it is equal to the advancing angle, Θ_A_.

Young’s law states that the contact angle between a liquid–air interface and an ideal solid is given by^1^
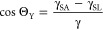
1where Θ_Y_ is Young’s contact angle, and γ_SA_, γ_SL_, and γ are the solid–air, solid–liquid, and liquid–air interfacial tensions, respectively. Since γ_SA_, γ_SL_, and γ are constant material properties for an ideal solid, eq 1 predicts that the contact angle is uniquely defined. Therefore, according to Young’s law, an ideal (solid) particle should be able to rotate freely at a liquid–air interface, as long as the interface has time to reach equilibrium (Figure 1a).

However, in reality, the (static) contact angle, Θ, does not take a single value, but it lies within a finite range, between the so-called receding and advancing contact angles (Θ_R_ ≤ Θ ≤ Θ_A_). A liquid only begins to advance relative to a solid when Θ ≥ Θ_A_, and it only begins to recede when Θ ≤ Θ_R_. This effect is called contact angle hysteresis. Contact angle hysteresis is caused by inhomogeneities on the surface of the solid and by the adaption of the solid to the liquid.^2−5^ All real solids (including particles) display contact angle hysteresis. Therefore, to rotate a particle relative to a liquid–air interface, the contact angle on the side that rolls out of the liquid must be equal to Θ_R_, whereas the contact angle on the side that rolls into the liquid must be equal to Θ_A_ [Figure 1b].

The influence of contact angle hysteresis on the rotation of particles at an interface is still largely unexplored, despite its potential relevance in addressing practical questions such as what causes granular matter (e.g., sand grains) to flow more slowly when moist? Dry particles easily roll and slide relative to one another.^6−8^ Unlike dry particles, moist particles are connected by microscopic liquid bridges.^9−12^ Particles connected to liquid bridges cannot roll and slide easily. Several mechanisms have been identified to explain why this is so. First of all, the liquid bridges cause an increase in adhesion between the particles,^13^ which results in an increase in friction.^14^ Second, the bridges form an extended network, resulting in a stiff structure.^15^ A third contribution is due to viscous dissipation within the liquid bridges.^9^ However, none of these contributions considers the influence of contact angle hysteresis on the ease with which particles can roll, even though contact angle hysteresis can significantly alter rolling friction, as shown by Schade and Marshall (2011)^16^ and Marshall (2014),^17^ who considered a particle rolling on a thin liquid film.

In this paper, we derive a general analytical expression for the resistive torque experienced by a particle rotating at an interface (Figure 1b). Surface tension always acts parallel to an interface. Therefore, on both the right and left sides of the particle, the surface tension vector has a component tangential to the particle. This tangential component produces a torque that opposes the rotation. Since the torque is caused by surface tension, we will call it capillary torque. In general, the capillary torque increases with contact angle hysteresis and has a maximum of the order of γ*RL*, where γ is the surface tension, *R* is the radius of the particle, and *L* is the diameter of the three-phase contact line around the particle. Our results demonstrate that contact angle hysteresis is an important factor that can severely restrict rotation at an interface when the magnitude of the torque causing rotation is ≪γ*RL*.

## Theory

### General Expression for the Torque

**Figure 2:**
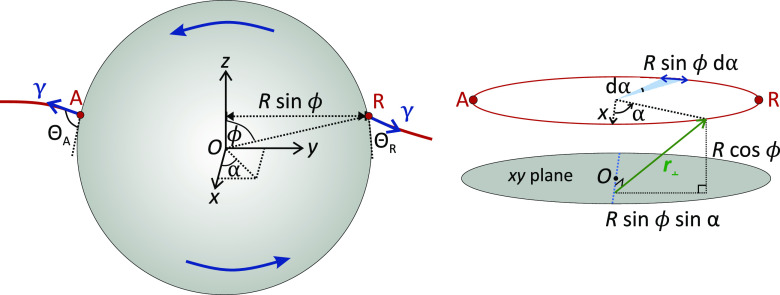
Particle rotating at an interface. Left: schematic of the particle rotating about a horizontal axis going through its center. The contact line is marked by points A and R. Right: the circular contact line is drawn in red. The blue dotted line shows the rotational axis.

As a model system, we consider a spherical particle at a liquid–fluid interface. In general, the second fluid can be any gas or liquid that is immiscible with the first liquid. In the following, we will refer to the second fluid as “air”. Our aim is to calculate the torque required to rotate the particle about the *x*-axis that goes through its center (Figure 2).^44^ When the particle rotates counterclockwise, the liquid–air interface recedes (advances) on the right (left) side of the axis of rotation. This asymmetry gives rise to a torque about the axis of rotation
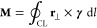
2where **r**_⊥_ is the perpendicular vector from the rotational axis to the contact line, × denotes the vector cross product, and d*l* = *R* sin* *ϕ dα is the contact line length element. The contour integral is around the contact line (CL), which we assume to be circular. **γ** acts at the contact line and makes an angle Θ(α) with the surface of the particle, where Θ(α) is the contact angle at an azimuthal angle α. In spherical coordinates, **γ** is given by
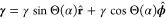
3where **r̂** is the radial unit vector from the center of the sphere and **ϕ̂** is the polar unit vector defined from the *z*-axis. **r**_⊥_ can most easily be expressed in terms of the Cartesian unit vectors
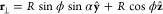
4Integrating eq 2 requires knowledge of the contact angle variation around the contact line, Θ(α). Θ(α) is not known for a rotating particle. However, we expect it to be analogous to contact angle variation around a drop moving on a flat surface. In both cases, there is relative motion between a solid and a liquid, with a receding contact angle on one side and an advancing contact angle on the opposite side. At the front of a moving drop, the contact angle corresponds to the advancing contact angle, whereas at the rear side, it corresponds to the receding contact angle. Several models have been proposed to describe the variation of the contact angle between these two extremities. Dimitrakopoulos and Higdon used a step function,^19^ Korte and Jacobi assumed Θ(α) to be linear in α,^20^ Extrand and Kumagai assumed that cos Θ(α) is linear in α,^21^ and ElSherbini and Jacobi demonstrated (experimentally) that both Θ(α) and cos Θ(α) can be fitted by a cubic polynomial.^22^ It turns out that the different assumptions lead to similar results, except for a different prefactor.

**Figure 3:**
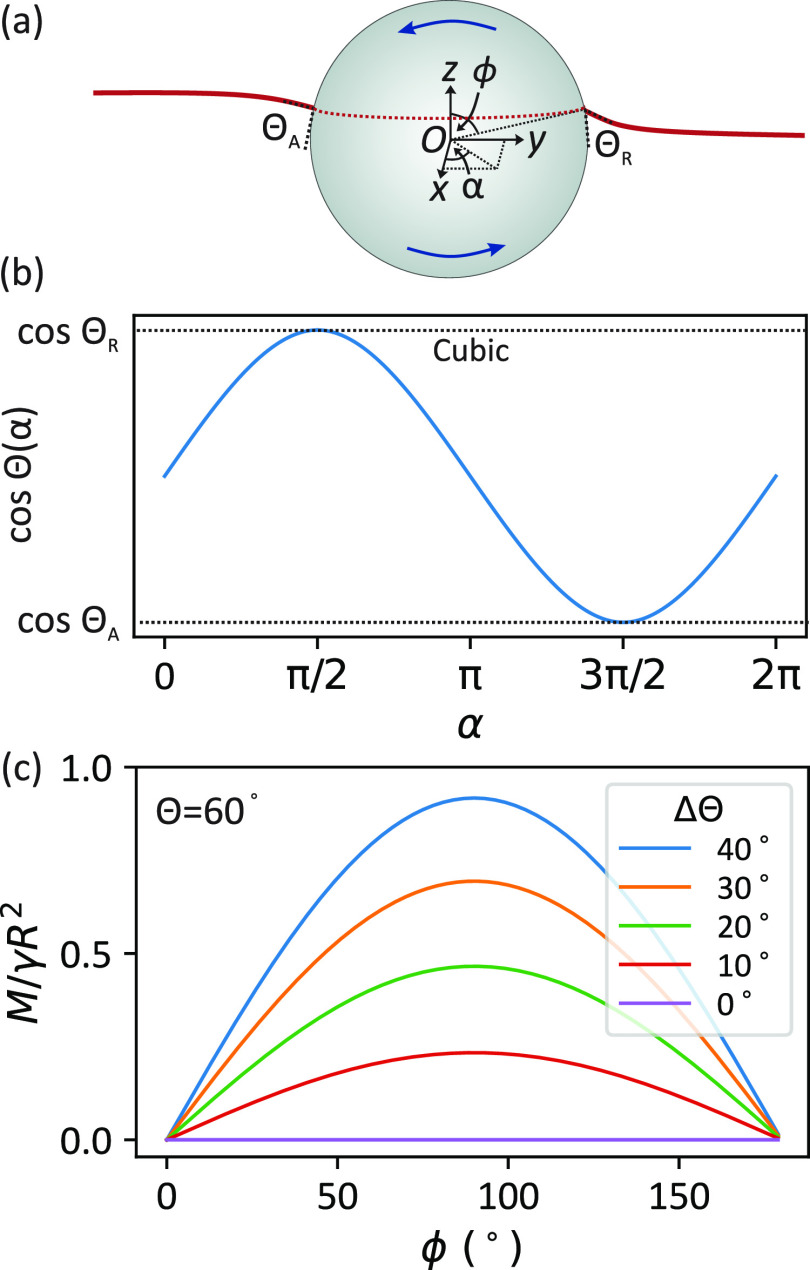
(a) Particle rotating at an interface. The contact line is assumed to be circular (dashed red). (b) Cos Θ(α) is assumed to follow a cubic polynomial in α. (b) Capillary torque acting on a sphere (average contact angle, Θ = (Θ_A_ + Θ_R_)/2 = 60°) as a function of polar angle, ϕ. The capillary torque increases with contact angle hysteresis, ΔΘ = Θ_A_ – Θ_R_, as shown by the different curves.

In the appendix, we evaluate eq 2 both for a step and a cubic variation in cos Θ(α) (Figure 3a) and obtain the following expression for the magnitude of the capillary torque
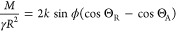
5where *k* = 1 for the step variation and *k* = 24/π^3^ ≈ 0.77 for the cubic variation (Supporting Information). Throughout the rest of this paper, we will use *k* = 24/π^3^ since the cubic variation allows the contact angle to vary in a realistic (smooth and continuous) way. The net capillary torque vector points along the *–x* direction (i.e., the torque acts clockwise) and therefore opposes the rotation. The *y* and *z* components of the capillary torque vector are zero due to the symmetry of the contact angle about the *yz* plane. We can also express eq 5 in terms of the average contact angle, Θ = (Θ_A_ + Θ_R_)/2, and the contact angle hysteresis, ΔΘ = Θ_A_ – Θ_R_
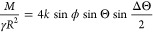
6Figure 3 (b) shows a plot of *M*/γ*R*^2^ as a function of ϕ, using Θ = 60° as an example. *M* has a maximum at ϕ = 90° because the length of the contact line is largest at this position. The torque tends to zero as ϕ tends to 0 and 180° since the length of the contact line goes to zero at these two extremities. As the contact angle hysteresis increases, the capillary torque also increases since a higher ΔΘ causes a more asymmetric interface.

## Results and Discussion

To gain further insights into the implications of the capillary torque, we consider two special cases: (1) when the particle rotates about its static equilibrium position and (2) when the particle is surrounded by a small liquid meniscus on a flat surface. Recent studies have argued that rotation is a relevant factor that needs to be considered when removing particles from surfaces by liquid–air interfaces (e.g., a drop).^23,24^ In the following, we quantitatively show that capillary torque is also important when describing particles in Brownian motion at an interface and when considering the rolling of wet particles on surfaces.

### Particle Rotating about its Equilibrium Position (ϕ = Θ)

**Figure 4:**
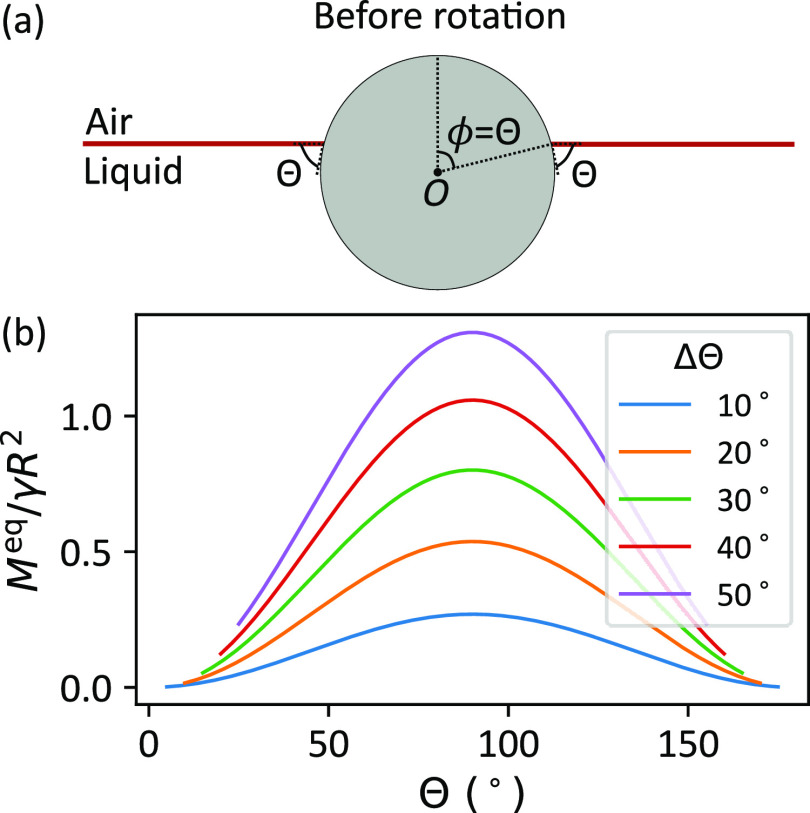
(a) Static particle in equilibrium at a liquid–air interface (ϕ = Θ). (b) Capillary torque as a function of average contact angle when the particle rotates about its initial equilibrium position.

This configuration (Figure 4a) is relevant to describe particles adsorbed at the surface of a lake, on the surface of a bubble (e.g., in flotation),^25^ or on the surface of droplets in a Pickering emulsion.^26,27^

When an external force is applied, the particle will not rotate unless the applied torque exceeds the maximum capillary torque. By substituting ϕ = Θ in eq 6, we obtain the capillary torque for a particle rotating about its equilibrium configuration
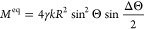
7*M*^eq^ is symmetric around Θ = 90° and it increases with contact angle hysteresis (Figure 4). We have restricted our results to a maximum contact angle hysteresis of ΔΘ = 50° since our assumptions about the shape of the contact line might no longer be appropriate for very large ΔΘ.

Practically, most particles are mildly hydrophilic to mildly hydrophobic (mean contact angle is approximately between 30° and 90° with water). Special treatments, such as plasma cleaning or the addition of nanoscale roughness, are usually required to achieve lower or higher average contact angles with water. Therefore, for most practical cases, the torque required to rotate a particle about its equilibrium position at an air–water interface is of the order of γ*R*^2^ (Figure 4b).

### Brownian Motion at an Interface

In thermal equilibrium, small particles exhibit Brownian motion. When particles are in Brownian motion at an interface, the translational motion is constrained to the two-dimensional interface.^28,29^ Furthermore, as we will show below, particles at an interface do not rotate as they would do when fully dispersed in the liquid. Rotation becomes negligible since it is opposed by capillary torque.

Here, we quantify this effect by calculating the root-mean-square angle through which thermal energy rotates a particle at an interface. As an example, we consider a hydrophobic particle with Θ = 90°, resting in equilibrium (half-submerged) at a horizontal air–water interface. In the complete absence of external forces, ϕ = Θ = 90°, along the entire contact line. When small rotational forces are applied, the contact line on the particle will remain pinned unless the angular rotation out of the plane of the interface is greater than half the contact angle hysteresis.

At room temperature, thermal energy will attempt to vibrate and rotate the particle. When thermal energy rotates the particle counterclockwise by a small angle ϑ, the contact angle on the right side becomes Θ – ϑ, and the contact angle on the left becomes equal to Θ + ϑ. Therefore, by substituting ΔΘ = 2ϑ (and Θ = 90°) in eq 7, we obtain the magnitude of the capillary torque resisting the thermal rotation as *M* = 4γ *k R*^2^ sin ϑ.

Since we anticipate ϑ to be small (an assumption that we will show to be valid below), we can write sin ϑ ≈ ϑ. The work required to overcome capillary torque and rotate the particle by ϑ about the *x*-axis is
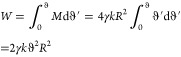
8As *W* is quadratic in ϑ, we can apply the equipartition theorem. According to the equipartition theorem, the thermal energy accessible to each rotational degree of freedom is *k*_B_*T*/2, where *k*_B_ is the Boltzmann constant and *T* is the absolute temperature. Since capillary torque influences rotation about the *x*- and *y*-axes, there are two degrees of freedom for rotation against the interface. Therefore, the average potential energy associated with rotating the particle against the interface is ⟨*W*⟩ = *k*_B_*T*. By equating ⟨*W*⟩ to eq 8, we obtain the root-mean-square angular displacement caused by Brownian motion
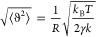
9For a nanoparticle with a radius of 50 nm (e.g., soot) at the surface of water (γ = 72 mN m^–1^ and *T* = 300 K), .^45^ For a 10 μm particle, the angle decreases to 0.01°. Since all real particles have a contact angle hysteresis much greater than these values, thermal energy is insufficient to overcome contact line pinning and cause the particles to rotate relative to the interface. Hence, thermal fluctuations will only be able to rotate nano- and microparticles by negligible amounts. Every time thermal fluctuations cause the particle to rotate, the pinned contact line will restore it back to the equilibrium configuration, thus preventing any continuous rotation.

### Particle Surrounded by a Meniscus

**Figure 5:**
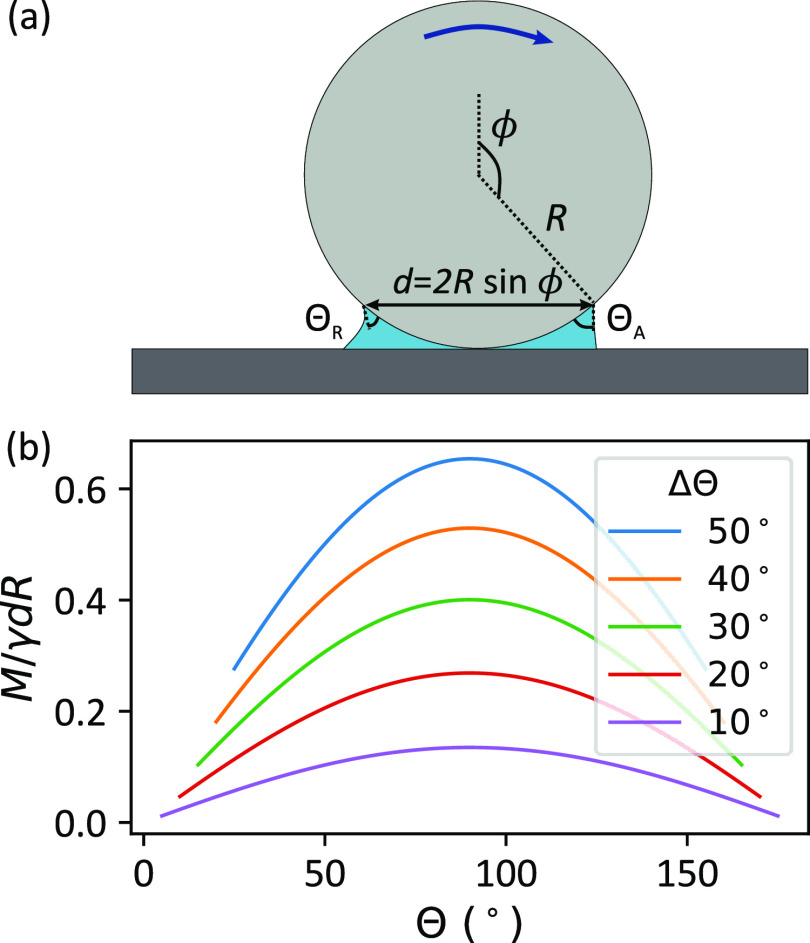
(a) Particle rolling on a flat surface with a liquid meniscus between the particle and the surface. (b) Capillary torque as a function of average contact angle, Θ = (Θ_A_ + Θ_R_)/2, for different contact angle hysteresis, ΔΘ = Θ_A_ – Θ_R_.

When a hydrophilic particle is placed in contact with a hydrophilic surface in air, a water meniscus forms around the contact region due to the condensation of water vapor from the atmosphere (Figure 5a).^13,30^ Capillary condensation leads to an increase in the normal adhesion force between particles and surfaces due to capillary forces acting through the water meniscus. The presence of a small meniscus between particles (e.g., moist sand grains) or between a particle and a flat surface also influences their rolling friction. One of the factors that contribute to the rolling friction is the capillary torque. When the contact line diameter between the particle and the meniscus is *d* (as sketched in Figure 5), the resistive capillary torque that needs to be overcome to roll the particle is obtained by substituting *d* = 2*R*  sin ϕ in eq 5
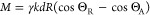
10This expression agrees with the expression derived by Schade and Marshall (2011) and by Marshall (2014),^16,17^ except for the prefactor. Marshall considered the torque about a single point at the center of the particle, rather than about the axis of rotation. In Figure 5b, *M*/γ*dR* (eq 10) is plotted against contact angle hysteresis for different average contact angles. We see that the capillary torque increases with contact angle hysteresis and is symmetric about Θ = 90°. For any contact angle hysteresis, the maximum corresponds to an average contact angle of 90° because in this case, the tangential component of surface tensions oppose rotation on both the advancing and receding sides. This is not the case for other values of Θ. For instance, when Θ_A_ = 50° and Θ_R_ = 30°, the tangential component of surface tension still opposes rotation on the receding side but acts in the direction of rotation on the advancing side. Therefore, the overall resistive torque is lower than when Θ = 90°.

Equation 10 is also valid for a particle rolling on a thin liquid film, like in the experiments performed by Bico et al.^31^ and Schade and Marshall (2011).^16^ However, in this case, capillary torque is only one of the several contributing factors to the resistive force acting on the particle. For a full description, the solid–solid rolling friction, which arises due to deformation losses and due to the energy required to peel the rear contact between the two solid surfaces,^32^ has to be included. Furthermore, the viscous forces and Laplace pressure distribution inside the meniscus have to be considered.^17^ The relative importance of each of these contributions depends on the material properties (viscosity, surface roughness, viscoelastic properties, and surface energies) of the particle, the flat substrate, and the liquid meniscus.^17^

Interestingly, capillary torque implies that the onset at which a particle begins to roll on a wet inclined surface occurs at a finite angle of inclination, even when there is no solid–solid rolling friction between the particle and the surface. To gain intuition on how significant the capillary torque is, we consider a particle on a flat surface tilted by an angle α to the horizontal. Our aim is to calculate how large the particle has to be for it to begin rolling down the inclined surface. We assume that the contact lines between the meniscus and the particle and between the meniscus and the flat surface remain pinned until rolling starts. The onset of rolling occurs when the driving torque due to the particle’s weight becomes equal to the capillary torque. The torque produced by the weight of the particle is *mgR*  sin α, where m is the mass, *g* = 9.81 m s^–2^ is the gravitational acceleration, and *R* is the radius of the particle, whereas the capillary torque is given by eq 10. Rolling only starts when
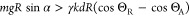
11
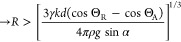
12where we have expressed *m* in terms of the volume of the particle (4π*R*^3^/3) and its density, ρ. As an example, we consider a glass particle (ρ ≈ 2500 kg m^–3^) surrounded by a small water meniscus with *d* = *R*/5, Θ_A_ ≈ 45°, and Θ_R_ ≈ 10°,^33^ on a surface tilted by α = 30°. A particle with these parameters only starts rolling if its radius is larger than ≈250 μm. Even though we have assumed that the capillary torque is the only source of resistance in this example, we still obtain a radius that is larger than the radius above which a dry particle would usually start rolling down a dry flat surface (a dry 100 μm glass bead easily rolls down an inclined glass slide).

Capillary torque could also be a significant factor that contributes to reducing the mobility of humid granular matter. Dry granular matter flows easily, as exemplified by sand flowing in an hourglass. In contrast, humid sand hardly flows and can even be molded into various stable structures, such as sandcastles.^34^ It appears that so far, capillary torque has not been considered when modeling humid granular matter.

### Unifying the Results

**Figure 6:**
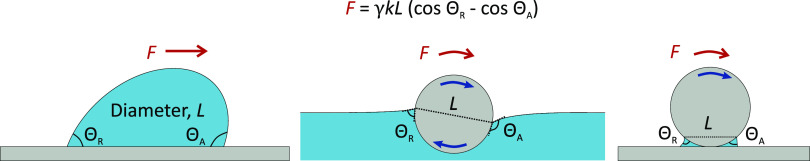
Equivalent scenarios. The same expression describes the force required to (1) move a drop on a flat surface, (2) rotate a particle at an interface, and (3) initiate the rolling of a particle on a flat surface when there is a liquid meniscus between the particle and the surface.

The expression for the capillary torque acting on a particle rotating at the surface of a liquid (eq 5) is similar to that for the capillary torque acting on a particle surrounded by a small meniscus on a flat surface (eq 10). Interestingly, when normalized by the contact line diameter and the particle’s radius, these expressions are equivalent to the expression describing the friction force (per unit diameter) experienced by a drop moving on a flat surface.^19−21,35−43^ For all three cases (Figure 6), the effective force is
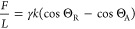
13where *L* is the diameter of the contact line and *k* = 24/π^3^ for a cubic contact angle variation. The prefactor *k* may vary depending on the precise contact line geometry and contact angle variation. In the case of the rotating particles, the effective force given by eq 13 corresponds to a force applied tangentially along the circumference of the particle.

We can take advantage of the similarity of the scenarios sketched in Figure 6 to indirectly determine the capillary torque (experimentally). Several methods have been developed to measure drop friction on various surfaces. In contrast, it is unusual, as well as practically challenging, to measure the torque required to rotate a small particle at an interface. Therefore, an estimate for the capillary torque that a particle made of material B would experience when it rotates at the interface between liquid A and air can be conveniently obtained by instead measuring the force required to move a drop of liquid A on a flat surface of material B.

## Conclusions

We have investigated the capillary forces acting on a particle when it rotates at an interface. We showed that a particle rotating at an interface experiences a resistive capillary torque. The larger the contact angle hysteresis, the greater the capillary torque. The expression for the capillary torque is similar to the expression for the friction force between a moving drop and a flat surface.

Our theory predicts that even for very small (nano/micro) particles, the energy required to overcome the capillary torque is much larger than thermal energy. Therefore, particles moving at an interface due to thermal energy do not rotate. Furthermore, capillary torque may be an important factor that needs to be included when modeling the flow of moist granular matter.

## Evaluation of Capillary Torque

To evaluate the cross product in eq 2, we express **γ** in Cartesian coordinates using the transformation

14

After applying the transformation to eq 3, we obtain **γ** in terms of the Cartesian unit vectors

15

16

17

18

Integrating the ***x*^** component of **r**_⊥_ × ***γ*** around the contact line (eq 2) gives

19

The *ŷ* component of the integral is

20

*M*_*y*_ evaluates to zero because the terms in α are sin Θ(α) cos α and cos Θ(α) cos α, which both evaluate to zero when integrated from 0 to 2π. These terms integrate to zero because sin Θ(α) and cos Θ(α) are even functions about the *yz* plane, whereas cos α is an odd function.

Hence, overall, the integrand is an odd function and therefore integrates to zero.

The *ẑ* component of the integral is

21

*M*_*z*_ evaluates to zero because the terms in α are sin Θ(α) sin 2α and cos Θ(α) sin 2α, which are both odd functions about the *yz* plane. Intuitively, *M*_*y*_ = 0 and *M*_*z*_ = 0 are expected due to the symmetry of the surface tension vector about the *yz* plane. Any surface tension component that produces a moment along the +*y* or +*z* direction is canceled by an equal and opposite component pointing along −*y* or −*z*, respectively. Thus, the only nonzero torque component is *M*_*x*_. Since the capillary torque opposes rotation, we expect *M*_*x*_ to be negative. As we are interested in the magnitude of the torque, we will consider −*M*_*x*_, which is positive. In the following, we will also drop the subscript and simply refer to −*M*_*x*_ as *M*.

For simplicity, we first consider a circular contact line divided between an advancing and a receding side with the following contact angle dependence

22

For this case, eq 19 (without the negative sign) can be written as

23

When a more realistic and complex expression is used to describe Θ(α), the resulting expression is similar to eq 23, but with a different prefactor

24

When cos Θ(α) is given by a cubic polynomial in α, we obtain *k* = 24/π^3^ (Supporting Information).

Alternatively, we can write eq 24 as

25

where Θ = (Θ_A_ + Θ_R_)/2 is the mean contact angle and ΔΘ= Θ_A_ – Θ_R_ is the contact angle hysteresis. The following trigonometric identity was used to arrive at eq 25 from eq 24

26

## Supporting Information Available

Calculation of the *k* factor in eq 5 (PDF)
