# Supplementary material for: Capillary Torque on a Particle Rotating at an Interface
Source: Langmuir. 2021 Jun 11;37(24):7457–63. doi: 10.1021/acs.langmuir.1c00851 (PMC8280771; doi:10.1021/acs.langmuir.1c00851)
Supplement: Supplementary file 1 — la1c00851_si_001.pdf [file la1c00851_si_001.pdf]

Capillary torque on a particle rotating at an interface

Abhinav Naga, Doris Vollmer, and Hans-Jürgen Butt

Max Planck Institute for Polymer Research,

Ackermannweg 10, 55128 Mainz, Germany

May 27, 2021

Supporting Information

Calculation of k factor for a cubic variation of $\cos\Theta(\alpha)$

In this section, the k factor in Eq. 6 is evaluated using a cubic function to describe $\cos\Theta(\alpha)$.

The contact angle function has to satisfy four boundary conditions in order to be both smooth and continuous:

- $\cos\Theta(\alpha = -\pi/2) = \cos\Theta_{\mathrm{A}}$,
- $\cos\Theta(\alpha = \pi/2) = \cos\Theta_{\mathrm{R}}$,
- $\frac{d(\cos\Theta)}{d\alpha} = 0$ at $\alpha = -\pi/2$, and
- $\frac{d(\cos\Theta)}{d\alpha} = 0$ at $\alpha = \pi/2$.

The last two conditions are required in order to have a smooth contact angle variation at the extremities. Since there are four boundary conditions, the function describing the contact

angle variation must have four independent parameters. A cubic polynomial fulfills this requirement:

$$\cos\Theta(\alpha) = a\alpha^3 + b\alpha^2 + c\alpha + d, \tag{S1}$$

where a, b, c, and d are independent variables. To find these variables in terms of the advancing and receding angles, we use the four boundary conditions listed above and obtain,

$$a = \frac{2}{\pi^3}(\cos\Theta_\mathrm{A} - \cos\Theta_\mathrm{R}) \tag{S2}$$

$$b = 0 \tag{S3}$$

$$c = -\frac{3}{2\pi}(\cos\Theta_\mathrm{A} - \cos\Theta_\mathrm{R}) \tag{S4}$$

$$d = \frac{1}{2}(\cos\Theta_\mathrm{A} + \cos\Theta_\mathrm{R}). \tag{S5}$$

Therefore, the equation describing $\Theta(\alpha)$ is

$$\cos\Theta(\alpha) = \frac{2}{\pi^3}(\cos\Theta_\mathrm{A} - \cos\Theta_\mathrm{R})\alpha^3 - \frac{3}{2\pi}(\cos\Theta_\mathrm{A} - \cos\Theta_\mathrm{R})\alpha + \frac{1}{2}(\cos\Theta_\mathrm{A} + \cos\Theta_\mathrm{R}). \tag{S6}$$

This equation is valid for $-\pi/2 < \alpha < \pi/2$.

The expression for the magnitude of the capillary torque is (negative of Eq. 19 from the Appendix in the main manuscript):

$$M = \gamma R^2 \sin\phi \int_0^{2\pi} \cos\Theta(\alpha)\sin\alpha \; d\alpha \tag{S7}$$

$$= \gamma R \frac{L}{2} \int_0^{2\pi} \cos\Theta(\alpha)\sin\alpha \; d\alpha, \tag{S8}$$

where R is the radius of the particle and $L = 2R\sin\phi$ is the diameter of the contact line.

To find the k factor corresponding to a cubic variation in $\cos\Theta(\alpha)$, we first rewrite the integral in Eq. S8 such that the limits of integration match the range for which our definition of $\cos\Theta(\alpha)$ is valid ($-\pi/2$ to $\pi/2$),

$$\begin{aligned}\frac{M}{\gamma RL} &= \frac{1}{2}\int_{0}^{2\pi} \cos\Theta(\alpha)\sin\alpha\ d\alpha \\ &= \frac{1}{2}\int_{-\pi}^{\pi} \cos\Theta(\alpha)\sin\alpha\ d\alpha \\ &= \int_{-\frac{\pi}{2}}^{\frac{\pi}{2}} \cos\Theta(\alpha)\sin\alpha\ d\alpha \end{aligned} \tag{S9}$$

The last step is due to symmetry of $\Theta(\alpha)$ about the yz plane.

Next, we substitute Eq. S1 into Eq. S9 to obtain

$$\begin{aligned}\frac{M}{\gamma RL} &= \int_{-\frac{\pi}{2}}^{\frac{\pi}{2}} \sin\alpha\left[a\alpha^3 + c\alpha + d\right]\ d\alpha \\ &= \left[\left(\frac{3\pi^2}{2} - 12\right)a + 2c + 0\right] \\ &= \frac{24}{\pi^3}(\cos\Theta_{\mathrm{R}} - \cos\Theta_{\mathrm{A}}). \end{aligned} \tag{S10}$$

Therefore, $k = 24/\pi^3$. The following results were used to perform the integration:

$$\begin{aligned}\int_{-\frac{\pi}{2}}^{\frac{\pi}{2}} \alpha\sin\alpha\ d\alpha &= \int_{-\frac{\pi}{2}}^{\frac{\pi}{2}} \alpha\frac{d(-\cos\alpha)}{d\alpha}\ d\alpha \\ &= [-\alpha\cos\alpha]_{-\frac{\pi}{2}}^{\frac{\pi}{2}} - -\int_{-\frac{\pi}{2}}^{\frac{\pi}{2}} \cos\alpha\ d\alpha \\ &= 2, \end{aligned}$$

and

$$
\begin{aligned}
\int_{-\frac{\pi}{2}}^{\frac{\pi}{2}} \alpha^3 \sin\alpha \ d\alpha &= \int_{-\frac{\pi}{2}}^{\frac{\pi}{2}} \alpha^3 \frac{d(-\cos\alpha)}{d\alpha} \ d\alpha \\
&= \left[-\alpha^3 \cos\alpha\right]_{-\frac{\pi}{2}}^{\frac{\pi}{2}} - -3 \int_{-\frac{\pi}{2}}^{\frac{\pi}{2}} \alpha^2 \cos\alpha \ d\alpha \\
&= 3 \int_{-\frac{\pi}{2}}^{\frac{\pi}{2}} \alpha^2 \frac{d(\sin\alpha)}{d\alpha} \ d\alpha \\
&= 3 \left[\alpha^2 \sin\alpha\right]_{-\frac{\pi}{2}}^{\frac{\pi}{2}} - 3 \int_{-\frac{\pi}{2}}^{\frac{\pi}{2}} 2\alpha \sin\alpha \ d\alpha \\
&= \frac{3\pi^2}{2} - 12.
\end{aligned}
$$
